# Hippocampal Malrotation Could Be Less Significant in Epilepsy Caused by Focal Cortical Dysplasia Type I and Type II

**DOI:** 10.3389/fneur.2022.755022

**Published:** 2022-02-14

**Authors:** Chenmin He, Lingqi Ye, Cong Chen, Lingli Hu, Bo Jin, Yao Ding, Hong Li, Meiping Ding, Shan Wang, Shuang Wang

**Affiliations:** ^1^Epilepsy Center, Department of Neurology, Second Affiliated Hospital, School of Medicine, Zhejiang University, Hangzhou, China; ^2^Department of Neurology, Zhejiang Provincial People's Hospital, Hangzhou, China; ^3^Epilepsy Center, Department of Radiology, Second Affiliated Hospital, School of Medicine, Zhejiang University, Hangzhou, China

**Keywords:** hippocampal malrotation, epilepsy, focal cortical dysplasia, seizure, MRI

## Abstract

**Objectives:**

Debates over the relationship between hippocampal malrotation (HIMAL) and epilepsy continue without consensus. This study explores the role of HIMAL in a cohort of epilepsy caused by focal cortical dysplasia (FCD).

**Methods:**

In this study, 90 patients with epilepsy caused by FCD type I and type II and 48 healthy adults underwent a 3 Tesla MRI following a dedicated epilepsy protocol for the analysis of the prevalence and morphologic features of HIMAL. In addition, numerous clinical characteristics and hippocampal volumes were evaluated.

**Results:**

The cohort included a total of 90 patients (32 were HIMAL, 58 were non-HIMAL). Among these patients, 32 (35.6%) had HIMAL (22 left, four right, and six bilateral), which did not differ from the 48 controls, where 16 (33.3%) had HIMAL (12 left, two right, and two bilateral). Neither the quantitative features of HIMAL (diameter ratio, dominant inferior temporal sulcus height ratio, medial distance ratio, dominant inferior temporal sulcus angle, and parahippocampal angle), nor the accompanying characteristics of HIMAL (vertical dominant inferior temporal sulcus, enlarged temporal horn, and a low position of ipsilateral fornix) showed differences between patients with FCD and controls. No statistical difference in the clinical characteristics between FCD patients with HIMAL and those without was found. Neither the side nor the existence of HIMAL was correlated with the lateralization and location of FCD. As to the hippocampal volume, there was no difference between FCD patients with HIMAL and those without.

**Conclusion:**

Hippocampal malrotation is a common morphologic variant in healthy controls as well as in patients with epilepsy caused by FCD type I and type II. Hippocampal malrotation could be less significant in epilepsy caused by FCD type I and type II.

## Introduction

Hippocampal malrotation (HIMAL), also described as incomplete hippocampal inversion, often presents with a round or pyramidal shape, medial position close to the midline, vertical collateral sulcus, and a low position of ipsilateral fornix (1–3). Recently, the relationship between HIMAL and epilepsy has been strongly contested. Some studies reported that HIMAL is an abnormal developmental sign in patients with epilepsy (1, 3, 4), while others suggested that HIMAL is a normal anatomic variant common in both patients with epilepsy and controls (2, 5). To date, the significance of HIMAL in epilepsy remains controversial.

Due to the similar appearance of HIMAL and fetal hippocampus at 14–20 weeks of gestation, some scholars speculate that HIMAL is the consequence of incomplete infolding of medial temporal structures during embryonic brain development, and could be a marker of developmental brain disorders (2, 6). It has been reported that patients with periventricular nodular heterotopia, lissencephaly, polymicrogyria, holoprosencephaly, and hemimegalencephaly often have HIMAL (4, 7–11), and eventually present epilepsy. Although HIMAL has not been directly considered as an epileptogenic lesion, it may be a factor of susceptibility to neuropathological processes which lead to the hippocampal neuronal loss and hippocampal sclerosis (12–14). Focal cortical dysplasia (FCD), a major cause of intractable epilepsy, was previously found to coexist with hippocampal abnormalities (7, 8). In patients with FCD type IIIa, the FCD is a developmental lesion left over from the fetal stage, whereas hippocampal sclerosis is a postnatally acquired lesion (15). In patients with FCD type II or type I, it is unclear whether HIMAL provides localization value or has any relationship with the epileptogenic zone. FCD patients with HIMAL during the presurgical evaluation are not uncommon. Additionally, most research on HIMAL have involved patients with malformation of cortical development (4, 7, 8), and little is known about the involvement of HIMAL in patients with FCD. Therefore, under this scenario, addressing the relationship between HIMAL and FCD can contribute to clinical work and fill in knowledge gaps.

To better understand the relationship between HIMAL and FCD in patients with epilepsy, we have compared the occurrence and morphologic features of HIMAL between epileptic patients with FCD and healthy controls. In addition, we systematically analyzed clinical characteristics as well as hippocampal volumes of FCD patients both with and without HIMAL.

## Materials and Methods

### Participants and Data Collection

We retrospectively reviewed the records of consecutive epileptic patients with FCD who were admitted to the Epilepsy Center of our hospital between 2013 and 2020. The inclusion criteria were: (1) patients with focal epilepsy; (2) MRI or postoperative pathology was designated as FCD type I and type II. The exclusion criteria were: (1) patients without the 3 Tesla MRI protocol of three-dimensional sagittal T1-weighted brain volume imaging (3D BRAVO); and (2) unsatisfactory imaging quality (subject movement or technical artifacts). In total, 90 patients with FCD were enrolled (22 were L-HIMAL, four were R-HIMAL, six were Bi-HIMAL, and 58 were non-HIMAL). All patients included in the study were born at term. Additionally, 48 healthy adult volunteers with no history of seizures were enrolled as controls (12 were L-HIMAL, two were R-HIMAL, two were Bi-HIMAL, and 32 were non-HIMAL). Controls were informed of the purpose and process of the study before the examination. This study was approved by the institutional review boards of the Second Affiliated Hospital of Zhejiang University.

Demographic and clinical data were collected, such as gender, age at seizure onset, duration of epilepsy, whether had aura or not, whether it was pharmacoresistant epilepsy or not, seizure frequency, history of febrile seizures, history of focal to bilateral tonic-clonic seizure, side of FCD lesion, location of FCD, and MRI imaging. All abnormalities based on MRI were re-reviewed by expert epilepsy-imaging neuroradiologists. MRI revealed several indicative features of FCD, such as local cortical thickening, blurring of the gray-white matter interface, and (with T2-weighted imaging) an increased focal signal of the subcortical white matter which often tapered toward the underlying ventricle (16). FCD lesion was categorized as either small lesion or large lesion according to the volume threshold of 3,217 mm^3^, as described in a previous study (17). Among patients with resective surgery, we evaluated the seizure outcome according to the last visit or telephone interview and classified them as either seizure-free (Engel class Ia) or non-seizure-free (Engle class Ib-IV) (18).

### MRI Acquisition

High-resolution MRI images were acquired using a 3.0 Tesla scanner (Discovery MR750, GE Healthcare, IL, USA). 3D BRAVO was acquired as follows: repetition time = 8.2 ms; echo time = 3.2 ms; flip angle = 12 degrees; spin-lattice relaxation time = 450 ms; matrix size = 256 × 256; sagittal plane resolution = 0.47 mm × 0.47 mm; and 1.0 mm slice thickness with no inter-slice gap. Images are typically acquired in the sagittal plane with isotropic reformats in axial and coronal planes being automatically generated. In addition, we used the coronal reformats for image analysis.

### Evaluation of Hippocampal Morphology

**Figure 1:**
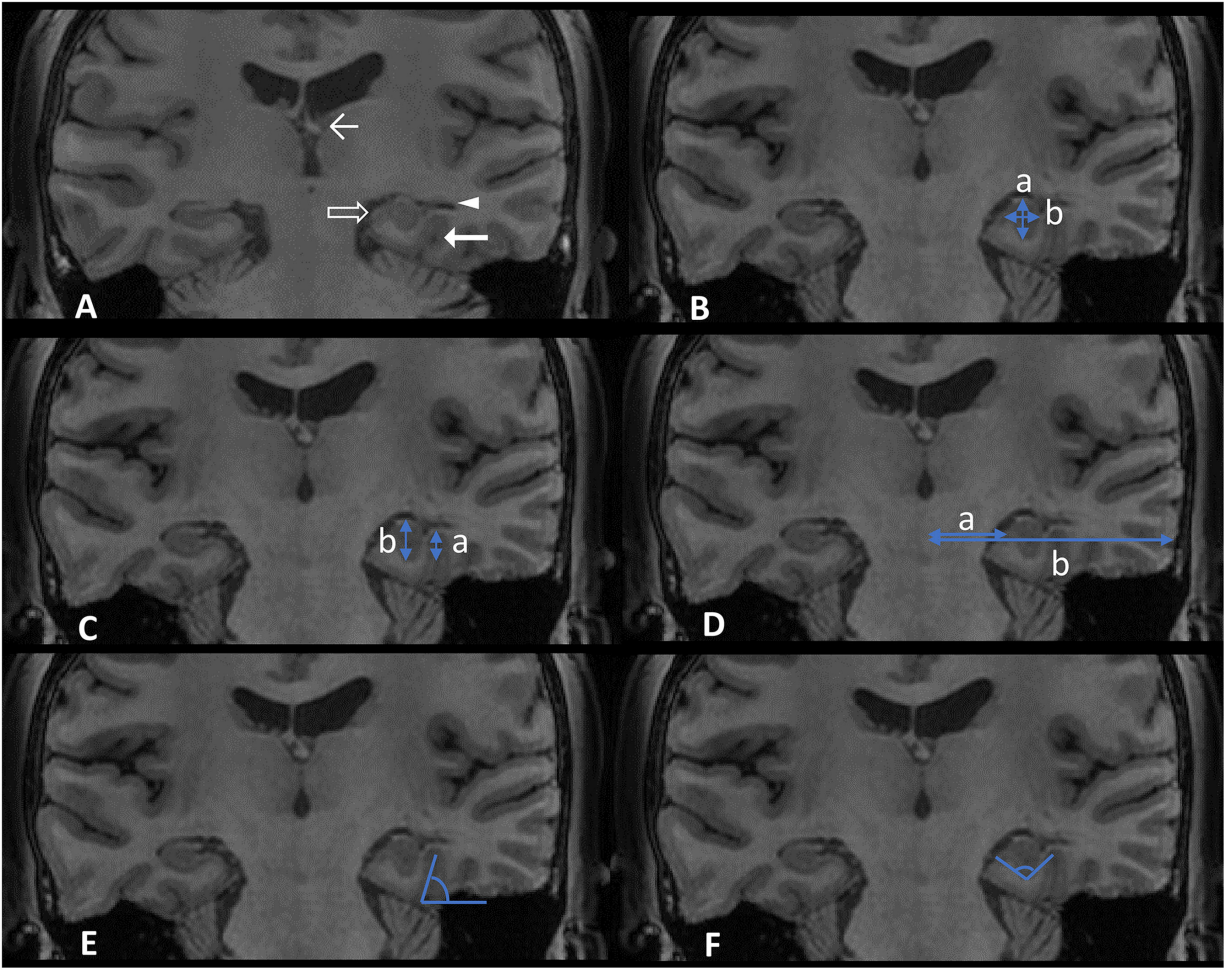
Features **(A)** and quantitative assessment **(B–F)** of hippocampal malrotation (HIMAL). **(A)** An example of left HIMAL as evidenced by a round shape (transparent arrow), vertical dominant inferior temporal sulcus (DITS) (thick arrow), enlarged temporal horn (triangle), and low position of ipsilateral fornix (thin arrow). **(B)** Hippocampal diameter ratio, a/b (blue arrow in **B**, a: height, b: width). **(C)** DITS height ratio, a/b (blue arrow in **C**). **(D)** Medial distance ratio, a/b (blue arrow in **D**). **(E)** DITS angle (blue angle in **E**). **(F)** Parahippocampal angle (blue angle in **F**).

We evaluated each hippocampus in four aspects (10): (1) round shape; (2) vertical dominant inferior temporal sulcus (DITS, the most prominent of the collateral or occipitotemporal sulcus); (3) enlarged temporal horn; and (4) a low position of ipsilateral fornix (Figure 1A). These four aspects were judged based on the comparison with the opposite side. Each hippocampus was defined as HIMAL, or non-HIMAL by the consensus of two investigators, where the round shape was the minimal criterion for the diagnosis of HIMAL. For the quantitative assessment of HIMAL, we chose the following previously defined features (2): (1) hippocampal diameter ratio (the hippocampal height divided by its width); (2) DITS height ratio (the height from the inferior margin of the hippocampus to the superior limit of the DITS, divided by the total hippocampal height); (3) medial distance ratio (the distance of the medial border of the hippocampus from the midline, divided by the distance of the lateral border of the temporal neocortex from the midline at the level of the temporal horn); (4) DITS angle (the angle of the DITS from the horizontal); and (5) parahippocampal angle (measured between the ascending and descending white matter segments of the parahippocampal gyrus) (Figures 1B–F).

### Evaluation of Hippocampal Volume

The 3D-T1 BRAVO images of all subjects were automatically segmented by Free Surfer software (v6.0.0, http://surfer.nmr.mgh.harvard.edu). The segmentation images were individually checked and (if necessary) manually corrected to obtain the volumes of the left and right hippocampus, as well as the total brain. The hippocampal volume was normalized by the intracranial volume.

### Statistical Analysis

The nonparametric Mann–Whitney *U*-test was used to compare group differences on continuous variables. Categorical variables were analyzed by Pearson's chi-square test or Fisher's exact test. The data of continuous variables were presented as median (percentiles 25–75%) by SPSS version 23.0. The significance level was set at *p* < 0.05. Interrater reliability was evaluated using Cohen's kappa correlation for qualitative assessments, and using intraclass correlation coefficient for quantitative assessments.

## Results

### HIMAL in Epileptic Patients With FCD and in Controls

Of the 104 patients meeting the inclusion criteria, two and 12 patients were excluded due to poor quality images and lacking 3D BRAVO, respectively. Collectively, a total of 90 patients were enrolled (42 men and 48 women). The median patient age was 16 years old [interquartile range (IQR) 10.75–25 years]. All the 90 patients included had no other lesions except FCD, and all FCD lesions were consistent with EEG (electroencephalogram) localization. Of these 90 patients, 32 (35.6%) had HIMAL (22 were left, four were right, and six were bilateral), and 58 (64.4%) had no HIMAL. Of the 48 controls, the median age was 30 years old (IQR 24–40 years). Furthermore, 16 (33.3%) had HIMAL (12 were left, two were right, and two were bilateral), and 32 (66.7%) had no HIMAL. There was no statistical difference in the prevalence between the two groups (*p* = 0.749).

As HIMAL occurred mostly on the left side (68.8% in patients with FCD and 75% in the controls), we compared the morphologic features of left-HIMAL (*L*-HIMAL) between the FCD group and control group (CTR). When performing statistical analysis, we used age as a covariable and determined that age did not affect the difference in quantitative features between the FCD group and the control group (hippocampal diameter ratio: *p* = 0.212; DITS height ratio: *p* = 0.546; medial distance ratio: *p* = 0.662; DITS angle: *p* = 0.432; parahippocampal angle: *p* = 0.342). The diameter ratio, DITS height ratio, medial distance ratio, DITS angle, and parahippocampal angle of the malrotated hippocampus all did not differ between the two groups. There was no statistical difference between the two groups in the four aspects of HIMAL (round shape, vertical DITS, enlarged temporal horn, and a low position of ipsilateral fornix). Only one FCD patient and two controls satisfied all four aspects of HIMAL (Table 1). Qualitative and quantitative assessments between the two investigators were similar (Tables 2, 3).

**Table 1:** The comparison in morphologic features of L-hippocampal malrotation (HIMAL) between the focal cortical dysplasia (FCD) and control (CTR) groups.

**Variables (%)**	**FCD (*n* = 22)**	**CTR (*n* = 12)**	***P*** **-value**
Round shape only	7 (31.8)	2 (16.7)	0.582^a^
Round shape +vertical DITS	3 (13.6)	1 (8.3)	1.000^a^
Round shape + a low position of ipsilateral fornix	3 (13.6)	0	0.537^b^
Round shape + enlarged temporal horn	0	2 (16.7)	0.118^b^
Round shape + vertical DITS + a low position of ipsilateral fornix	1 (4.5)	2 (16.7)	0.577^a^
Round shape +vertical DITS + enlarged temporal horn	1 (4.5)	2 (16.7)	0.577^a^
Round shape + a low position of ipsilateral fornix + enlarged temporal horn	6 (27.3)	1 (8.3)	0.389^a^
Round shape + vertical DITS + a low position of ipsilateral fornix + enlarged temporal horn	1 (4.5)	2 (16.7)	0.577^a^
Diameter ratio, median	1.10 (0.97–1.18)	1.07 (0.95–1.15)	0.403^c^
DITS height ratio, median	0.58 (0.44–0.67)	0.61 (0.41–0.68)	0.763^c^
Medial distance ratio, median	0.32 (0.29–0.34)	0.30 (0.27–0.33)	0.217^c^
DITS angle, median	63.47 (53.74–74.38)	71.06 (64.52–84.13)	0.074^c^
Parahippocampal angle, median	92.66 (85.45–101.03)	92.30 (78.83–99.11)	0.606^c^

*L, left; HIMAL, hippocampal malrotation; FCD, focal cortical dysplasia; CTR, control; DITS, dominant inferior temporal sulcus*.

a *Chi-square test, statistically significant difference (p < 0.05)*.

b *Fisher's exact test*.

c *Nonparametric Mann–Whitney U-test*.

**Table 2:** Inter-rater reliability of qualitative assessments for HIMAL.

**Variables (%)**	**Kappa coefficient**
Round shape of left hippocampus	0.721
Round shape of right hippocampus	0.606
Round shape of bilateral hippocampus	0.609
Vertical DITS of L-HIMAL	0.619
A low position of ipsilateral fornix of L-HIMAL	0.766
Enlarged temporal horn of L-HIMAL	0.548

*L, left; HIMAL, hippocampal malrotation; DITS, dominant inferior temporal sulcus*.

*The interpretation of Kappa coefficient: 0–0.2 indicates slight agreement, 0.21–0.4 indicates fair agreement, 0.41–0.6 indicates moderate agreement, 0.61–0.8 indicates “substantial” agreement, and >0.8 indicates “almost perfect” agreement*.

**Table 3:** Inter-rater reliability of quantitative assessments for HIMAL.

**Variables (%)**	**Intraclass correlation coefficient**
Diameter ratio of L-HIMAL	0.692
DITS height ratio of L-HIMAL	0.730
Medial distance ratio of L-HIMAL	0.866
DITS angle of L-HIMAL	0.466
Parahippocampal angle of L-HIMAL	0.910

*L, left; HIMAL, hippocampal malrotation; DITS, dominant inferior temporal sulcus*.

*The interpretation of intraclass correlation coefficient: 0–0.2 indicates poor agreement, 0.21–0.4 indicates fair agreement, 0.41–0.6 indicates moderate, 0.61–0.8 indicates strong agreement, and >0.8 indicates almost perfect*.

### Characteristics in FCD Patients With and Without HIMAL

Of 90 epileptic patients with FCD meeting the inclusion criteria, 42 (46.7%) were men. The median age at seizure onset was 7 years (IQR 3–11.25 years), and the median epilepsy duration was 7 years (IQR 4–14.25 years). Of the total 90 patients, 18 (20%) had aura, eight (8.9%) had febrile seizures, and 20 (22.2%) had a history of focal to bilateral tonic-clonic seizures. An FCD lesion was located in the left hemisphere in 46 patients (51.1%). The most frequent FCD location was frontal (64.4%), followed by parietal (13.3%), temporal (4.4%), occipital (5.6%), and insular (4.4%). Multilobar extension was present in seven patients (7.8%). FCD lesion was pathologically confirmed in 56 patients (51 were FCD type II and five were FCD type I), and the remaining 34 were diagnosed by MRI. Small FCD lesions were found in 62 of 90 patients (68.9%). Among the 56 patients undergoing resective surgery, 46 (82.1%) were seizure-free at the final follow-up.

**Figure 2:**
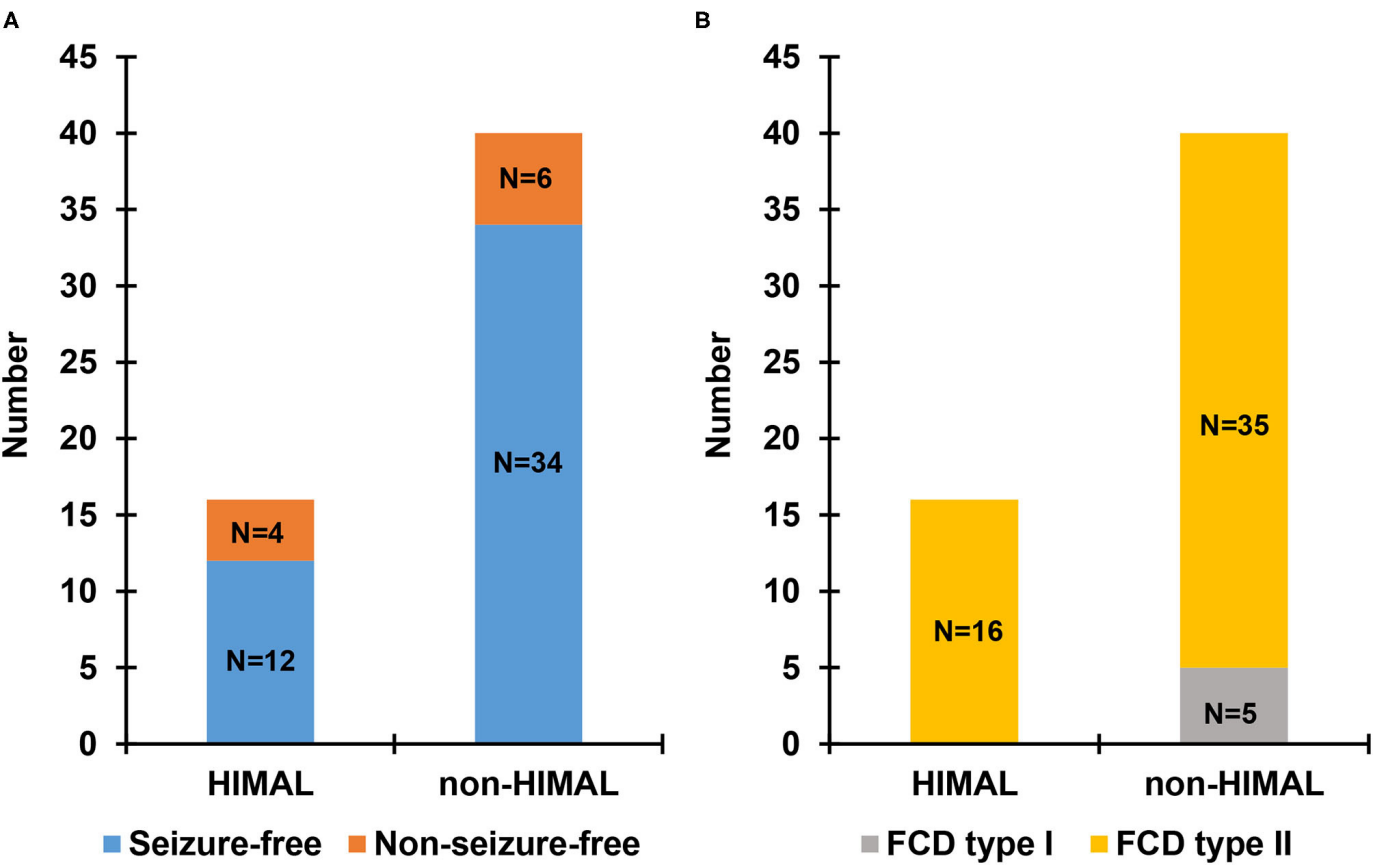
Seizure outcome **(A)** and pathologic type **(B)** in patients with focal cortical dysplasia (FCD) undergoing surgery.

There was no significant difference between the HIMAL group and the non-HIMAL group regarding gender, age at onset, epilepsy duration, aura, history of focal to bilateral tonic-clonic seizures, febrile seizures, FCD lesion size, or the location of FCD (Table 4). As shown in Table 5, HIMAL occurred more commonly on the left side in both left and right FCD, while there was no significant relationship between the side of HIMAL and FCD. Among the 90 patients with FCD, 56 patients underwent subsequent resective surgery, and there was no difference in the rate of seizure freedom among HIMAL and non-HIMAL groups (*p* = 0.620; Figure 2A). Additionally, no difference was found in the FCD types (*p* = 0.307; Figure 2B).

**Table 4:** Clinical characteristics of FCD patients with HIMAL and without HIMAL.

**Variables (%)**	**ALL (*n* = 90)**	**HIMAL** **(*n* = 32)**	**non-HIMAL** **(*n* = 58)**	***P*** **-value**
Gender, male	42 (46.7)	11 (34.4)	31 (53.4)	0.083^a^
Age at onset/year, median	7 (3–11.25)	8 (3.5–11)	6 (3–12)	0.614^c^
Epilepsy duration/year, median	7 (4–14.25)	8 (5–15.75)	6 (4–13.25)	0.327^c^
Pharmacoresistant epilepsy	49 (54.4)	14 (43.8)	35 (60.3)	0.130^a^
Aura	18 (20)	8 (25)	10 (17.2)	0.378^a^
Febrile seizure	8 (8.9)	4 (12.5)	4 (6.9)	0.612^a^
Seizure frequency, daily	41 (45.6)	12 (37.5)	29 (50)	0.254^a^
History of focal to bilateral tonic-clonic seizure	20 (22.2)	7 (21.9)	13 (22.4)	0.953^a^
Side of the FCD lesion, left	46 (51.1)	20 (62.5)	26 (44.8)	0.108^a^
MRI-positive	71 (78.9)	28 (87.5)	43 (74.1)	0.137^a^
Small FCD lesion	62 (68.9)	20 (62.5)	41 (70.7)	0.426^a^
**Location of FCD**				
Frontal lobe	58 (64.4)	20 (62.5)	38 (65.5)	0.775^a^
Temporal lobe	4 (4.4)	1 (3.1)	3 (5.2)	1.000^a^
Parietal lobe	12 (13.3)	5 (15.6)	7 (12.1)	0.880^a^
Occipital lobe	5 (5.6)	2 (6.3)	3 (5.2)	1.000^a^
Insular lobe	4 (4.4)	0	4 (6.9)	0.293^b^
Multilobe	7 (7.8)	4 (12.5)	3 (5.2)	0.406^a^

*FCD, focal cortical dysplasia; HIMAL, hippocampal malrotation*.

a *Chi-square test, statistically significant difference (p < 0.05)*.

b *Fisher's exact test*.

c *Nonparametric Mann–Whitney U-test*.

**Table 5:** Relationship between FCD side and HIMAL side.

**Variables**	**Left FCD (*n* = 20)**	**Right FCD (*n* = 12)**	***P*** **-value**
L-HIMAL (*n* = 22)	12	10	0.325^a^
R-HIMAL (*n* = 4)	2	2	1.000^a^
Bi-HIAML (*n* = 6)	6	0	0.061^b^

*FCD, focal cortical dysplasia; HIMAL, hippocampal malrotation; L, left; R, right; Bi, bilateral*.

a *Chi-square test, statistically significant difference (p < 0.05)*.

b *Fisher's exact test*.

**Figure 3:**
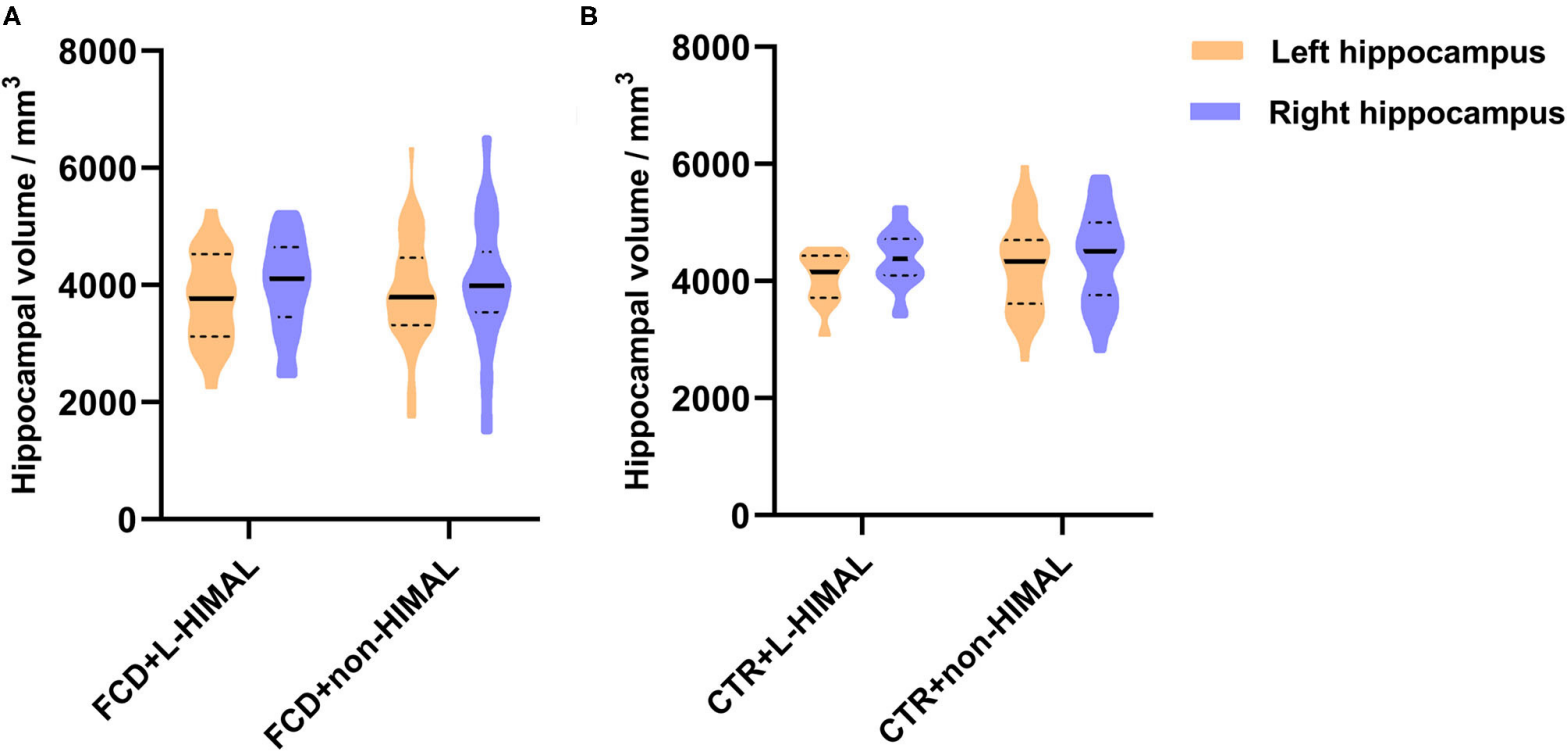
**(A)** Comparison of the hippocampal volume in FCD group and **(B)** CTR group. We compared the normalized hippocampal volume between the FCD + L-HIAML (*n* = 22) and FCD + non-HIMAL (*n* = 58) groups, where no difference was found (left hippocampal volume, *p* = 0.553; right hippocampal volume: *p* = 0.855; **A**). Additionally, no difference was found between the CTR + L-HIAML group (*n* = 12) and the CTR + non-HIMAL group (*n* = 32) (left hippocampal volume, *p* = 0.429; right hippocampal volume: *p* = 0.959; **B**).

We then compared the normalized hippocampal volume between the FCD + *L*-HIAML (*n* = 22) and FCD + non-HIMAL (*n* = 58) groups, where no difference was found (left hippocampal volume, *p* = 0.553; right hippocampal volume: *p* = 0.855; Figure 3A). Additionally, no difference was found between the CTR + *L*-HIAML group (*n* = 12) and the CTR + non-HIMAL group (*n* = 32) (left hippocampal volume, *p* = 0.429; right hippocampal volume: *p* = 0.959; Figure 3B).

**Figure 4:**
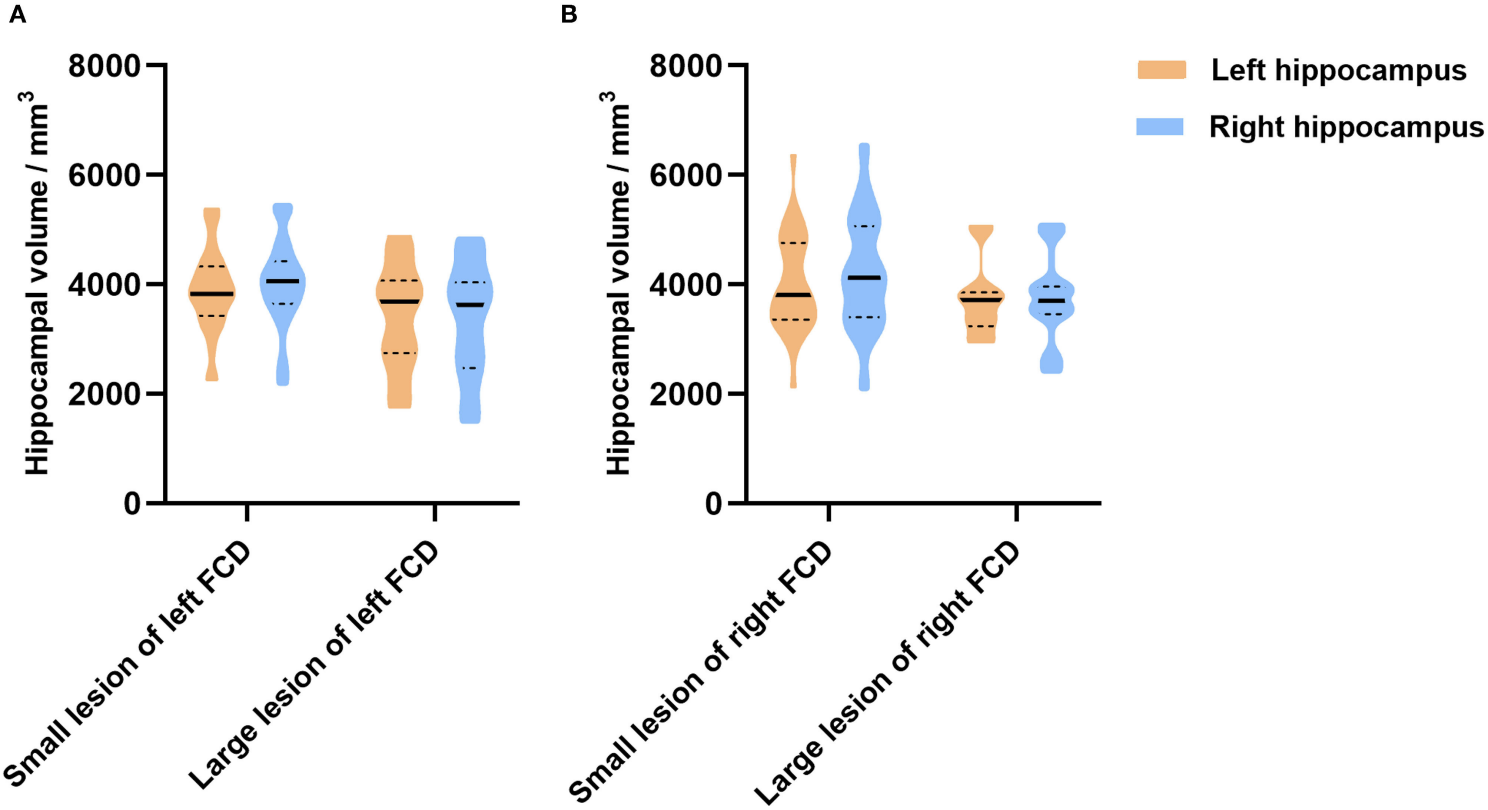
The relationship between the dimension of FCD and the hippocampal volume in extra-temporal lobe epilepsy. In patients with left FCD **(A)**, hippocampal volume (left, *p* = 0.168; right, *p* = 0.054) showed no difference between patients with small lesions and large lesions. In patients with right FCD **(B)**, hippocampal volume (left, *p* = 0.445; right, *p* = 0.155) showed no difference between patients with small lesions and large lesions as well.

In addition, we further analyzed whether the dimension of FCD influence the hippocampal volume. Due to the few cases with temporal lobe epilepsy (*n* = 4) and temporal plus epilepsy (*n* = 2), we focused on patients with extra-temporal FCD. Hippocampal volume showed no difference between small lesion and large lesion groups (Figures 4A,B).

## Discussion

In our study, we found a similar rate of HIMAL in patients with FCD (35.6%) and controls (33.3%). The prevalence of HIMAL in the controls of the previous studies (18–24%) was somewhat lower than what we found and may be explained by methodological issues, such as different samples, MRI protocols, and different diagnostic criteria (2, 19). A genome-wide association study on the genetics of HIMAL reported a positive correlation between the prevalence of HIMAL and intelligence/education attainment (20). Further is needed to better understand the significance of these findings.

The clinical characteristics did not differ between FCD patients with HIMAL and those without. In our epilepsy center, surgical decision-making in patients with FCD was made based on multidisciplinary discussions. For MRI-positive patients with FCD, if the lesion revealed by MRI was consistent with Video EEG (VEEG) recording, PET imaging, and semiology, the patient was recommended for directed surgery. If the imaging and electroclinical data were inconsistent, intracranial EEG was recommended to accurately localize the epileptogenic zone. For MRI-negative patients, intracranial EEG was also recommended. In patients who underwent surgery (*n* = 56), 46 patients (82.1%) were seizure-free until the most recent last follow-up, and the rate of seizure freedom showed no difference between the HIMAL group and the non-HIMAL group. The location of the HIMAL was not correlated with the lateralization of the epileptogenic zone, which is consistent with previous studies (2, 21). Additionally, a previous study (22) detected no relationship between the laterality of abnormal EEG and the laterality of HIMAL. In respect to the susceptibility of HIMAL persons to develop epilepsy, a systematic review found no significantly increased probability of epilepsy, suggesting that the presence of HIMAL should not be considered a strong independent predictor for epilepsy development (10). Based on the above, HIMAL seemed to have no tight relationship with epilepsy. However, any slight relationship between HIMAL and epilepsy is still unclear. In juvenile myoclonic epilepsy, HIMAL was identified in 51% of patients, and 50% of the unaffected siblings of those patients also had HIMAL (23). Chromosome 22q11.2 deletion syndrome, a 7-fold increased risk of developing seizures, was found to be associated with HIMAL in a population with neuropsychiatric disorders (24), and in this chromosomopathy, there often is a generalized micro-columnar architecture of the cortex similar to FCD type Ia (25). It was reported that pediatric epilepsy patients with HIMAL had no significant weakness of memory, but were attentionally impaired when performing more demanding dual tasks. It has been speculated that HIMAL is associated with complex prefrontal dysfunction (26), and future functional MRI studies of HIMAL may support this.

Human cortex development occurs at 15–24 weeks of gestation (27), and incomplete infolding of the hippocampus at 14–20 weeks (2). Developmental malformations of the brain can significantly affect hippocampal orientation. One previous study reported that the hippocampus was in the wrong position or was severely disoriented when telencephalic flexure (which forms the Sylvian fissure) was absent or abnormal (11). In lobar and semilobar holoprosencephaly, a telencephalic flexure does not form and the hippocampus does not “rotate” from a dorsal to ventral position and is more posterior in the hemisphere. This is similar to the mature rodent brain in which a telencephalic flexure and Sylvian fissure do not normally form (11). Hemimegalencephaly is considered to be an extensive FCD type IIb (28, 29). In hemimegalencephaly, the sylvian fissure is very distorted, as is the ipsilateral hippocampus (11). Epilepsy is present in ~40–60% of children with holoprosencephaly, whereas in hemimegalencephaly, the incidence is nearly 100% (30). In agenesis of the corpus callosum, the hippocampus may be disoriented, but epilepsy is infrequent (about 7–16%) (31, 32). HIMAL is common in patients with other malformation of cortical development, such as polymicrogyria and periventricular nodular heterotopia (5, 7, 8). In polymicrogyria, periventricular polymicrogyria is most likely to be associated with HIMAL since the development of the Sylvian fissure is affected prenatally. The prevalence of HIMAL in patients with malformation of cortical development is higher than in temporal lobe epilepsy, where its lateralization might not overlap with that of the epileptogenic focus, suggesting independent etiologies (2). This indicates that HIMAL may not be an entirely benign finding. However, we found no significant difference in the prevalence and features of HIMAL between patients with FCD and controls. Additionally, hippocampal volume in FCD patients with HIMAL and without HIMAL showed no difference. Our results indicate that FCD could be less associated with HIMAL, and larger samples are needed to confirm these findings in the future.

Previous studies reported that HIMAL occurred more commonly on the left side in both epilepsy patients and controls (2, 5, 26, 33, 34). This could result from an asynchronous developmental speed where the left hippocampus is much faster than the right during embryonic brain development (2).

The histopathology of HIMAL differs from that of hippocampal sclerosis (HS). The diagnostic hallmarks of HS are severe volume loss of the hippocampus, severe neuronal loss, and reactive gliosis involving two particularly vulnerable fields, CA1 and the subiculum (35). However, neuronal loss in HIMAL was less extensive than in HS (36). Previous studies have reported that postoperative examinations in HIMAL revealed the abnormal shape of the dentate gyrus and an atypical convolution of the CA1 pyramidal cell-subicular layers (37). Another study reported complex folding of the pyramidal cell layer of CA1 of HIMAL, which appeared excessively long and serpiginous (14). This indicates that changes in the CA1 region played an important role in HIMAL formation.

Although vertical DITS was always found in HIMAL, it was not unique to HIMAL and not specific for the diagnosis of HIMAL. It has been reported that vertical DITS was found in 21% of subjects with normal oval hippocampal shape (5), confirming the common variation of the DITS. In addition, there were various definitions of the vertical DITS in reported studies (5, 21, 33). One study defined vertical DITS as “> 70 degrees to the horizontal” (5), while another confirmed vertical DITS as “ <60 degrees from vertical” (21), thus complicating any comparison of vertical DITS among different studies. In this study, we found that both sides of DITS tended to be perpendicular in a small part of the FCD patients with unilateral HIMAL. This could result from the normal variation of DITS, and should not be considered as HIMAL accompanied by vertical DITS. Therefore, we adopted a method of bilateral comparison and defined vertical DITS as “the angle of the DITS from the horizontal on the HIMAL side being > 25 degrees larger than that of the opposite side”. In patients with HS, an enlarged temporal horn was a typical sign of hippocampal atrophy (38). In FCD patients with HIMAL, the temporal horn was not truly larger but only appeared larger due to the malrotated hippocampus, thus giving the appearance of a “small” hippocampus (8). Through the analysis of hippocampal volume, we confirmed that the malrotated hippocampus did not shrink in size. Another study found no significant relationships between whole hippocampal volumes and HIMAL, while the HIMAL severity was related to hippocampal subfield volumes, most notably the CA1 (12). Others suggested that the hippocampal segmentation of HIMAL might be altered by the malrotated hippocampus, making volumetric analysis inaccurate (20, 39). Because of these discrepancies, we did not compare the hippocampal subfield volumes among groups. The low position of ipsilateral fornix was prevalent in 58% of patients with other neurological diseases (such as, headache, cerebrovascular accident, transient ischemic attack, memory loss, and vertigo), but without seizures or HIMAL (3). We speculate that the fornix position might also be a normal morphological variant and represent the asymmetry in the development of the left and right brain. The judgment of the fornix's asymmetry could be influenced by the condition where the left and right sections of the fornix are not on the same plane on the MRI scan. Among the various characteristics, hippocampal shape change was the most reliable feature of HIMAL and was also the method commonly used in the clinical diagnosis of HIMAL using visual analysis (10). Some studies included the blurred structure of the hippocampus as a criterion (3, 31). Due to the subjectivity, we did not include this in our study. Consistent with us, a 7-T MRI study found that the internal architecture of the hippocampus is actually not blurred in patients with HIMAL (10, 40), and should probably not be considered a requirement for HIMAL diagnosis. In consideration of the above issues, criteria for a more scientific diagnostic of HIMAL are urgent.

## Limitations

The present study has some limitations. First, even though we manually checked and corrected the segmentation images, the presence of hippocampal anomalies can reduce the quality of these automatic brain segmentation methods. Second, there was a limited number of patients with FCD in temporal lobes, so we cannot analyze whether temporal FCD increased HIMAL risk. Third, the sample sizes of patients with FCD and controls were not large. Fourth, the evaluation of the signal intensity was not included in this study as some patients lacked a thin layer of T2 images in our cohort. Finally, reflecting the reality of everyday practice, not all patients with FCD included in our study underwent resective surgery.

## Conclusion

HIMAL is a common morphologic variant in healthy controls as well as in patients with epilepsy caused by FCD type I and type II. HIMAL could be less significant in epilepsy caused by FCD type I and type II.

## Data Availability Statement

The original contributions presented in the study are included in the article/supplementary material, further inquiries can be directed to the corresponding authors.

## Ethics Statement

The studies involving human participants were reviewed and approved by the Institutional Review Boards of The Second Affiliated Hospital of Zhejiang University. Written informed consent to participate in this study was provided by the participants' legal guardian/next of kin.

## Author Contributions

CH and ShuW contributed to the conception and design of the study. CH, LY, CC, LH, BJ, HL, and YD collected and analyzed the original data. ShaW and ShuW revised the manuscript. MD provides the technique and material support. All authors contributed to drafting the paper and were involved in the approval of the final version.

## Funding

This work was supported by the National Natural Science Foundation of China [Grant Numbers: 82001365, 82071443, and 81971208].

## Conflict of Interest

The authors declare that the research was conducted in the absence of any commercial or financial relationships that could be construed as a potential conflict of interest.

## Publisher's Note

All claims expressed in this article are solely those of the authors and do not necessarily represent those of their affiliated organizations, or those of the publisher, the editors and the reviewers. Any product that may be evaluated in this article, or claim that may be made by its manufacturer, is not guaranteed or endorsed by the publisher.
